# HRas and Myc synergistically induce cell cycle progression and apoptosis of murine cardiomyocytes

**DOI:** 10.3389/fcvm.2022.948281

**Published:** 2022-10-20

**Authors:** Aleksandra Boikova, Megan J. Bywater, Gregory A. Quaife-Ryan, Jasmin Straube, Lucy Thompson, Camilla Ascanelli, Trevor D. Littlewood, Gerard I. Evan, James E. Hudson, Catherine H. Wilson

**Affiliations:** ^1^Department of Pharmacology, University of Cambridge, Cambridge, United Kingdom; ^2^Department of Biochemistry, University of Cambridge, Cambridge, United Kingdom; ^3^QIMR Berghofer Medical Research Institute, Herston, QLD, Australia

**Keywords:** Myc (c-Myc), HRas gene, cardiomyocyte, proliferation, cell-cycle

## Abstract

**Aim:**

Adult mammalian cardiomyocytes are incapable of significant proliferation, limiting regeneration after myocardial injury. Overexpression of the transcription factor Myc has been shown to drive proliferation in the adult mouse heart, but only when combined with Cyclin T1. As constitutive HRas activity has been shown to stabilise Cyclin T1 *in vivo*, we aimed to establish whether Myc and HRas could also act cooperatively to induce proliferation in adult mammalian cardiomyocytes *in vivo*.

**Methods and results:**

Using a genetically modified mouse model, we confirmed that constitutive HRas activity (HRas*^G^*^12^*^V^*) increased Cyclin T1 expression. HRas*^G^*^12^*^V^* and constitutive Myc expression together co-operate to drive cell-cycle progression of adult mammalian cardiomyocytes. However, stimulation of endogenous cardiac proliferation by the ectopic expression of HRas*^G^*^12^*^V^* and Myc also induced cardiomyocyte death, while Myc and Cyclin T1 expression did not.

**Conclusion:**

Co-expression of Cyclin T1 and Myc may be a therapeutically tractable approach for cardiomyocyte neo-genesis post injury, while cell death induced by HRas*^G^*^12^*^V^* and Myc expression likely limits this option as a regenerative therapeutic target.

## Introduction

The adult heart is one of the least regenerative organs of the human body. Shortly after birth of a mammal, cardiomyocytes exit the cell cycle and are subsequently characterised by a reduced rate of turnover (<1% per year in humans) (1). Consequently, if the adult mammalian heart is damaged (e.g., myocardial infarction), the default response is to replace the lost cardiomyocytes with non-contractile fibrotic scar tissue and cardiomyocyte hypertrophy which frequently lead to heart failure. A number of strategies have been proposed to promote myocardial regeneration post damage, including the direct injection of stem cells or stem cell-derived cardiomyocytes, direct reprogramming of non-myocytes into cardiomyocytes and endogenous cardiomyocyte proliferation (2). Endogenous cardiomyocyte proliferation requires driving the resident quiescent cardiomyocytes into a productive mitogenic cell cycle. Genetic lineage tracing studies in the regenerative neonatal mouse and zebrafish hearts indicate that the majority of newly generated cardiomyocytes are derived from endogenous cardiomyocyte proliferation rather than differentiation from a mesenchymal progenitor (3–5). These studies suggest that forced cardiomyocyte proliferation is a viable strategy for myocardial regeneration and several potential factors capable of reactivating endogenous proliferation of cardiomyocytes in adult hearts have been identified, for example, inhibition of Hippo protein kinase signalling, enforced expression of cell cycle regulators, inactivation of thyroid hormone signalling, and hypoxia, all induce regeneration (6–10). We have recently shown that combined ectopic expression of Myc and Cyclin T1 can lead to extensive cardiomyocyte proliferation in the mouse heart (11), although exploitation of the therapeutic potential requires further exploration.

Myc is a pleiotropic transcription factor that, amongst other activities, regulates cell growth and proliferation in mammalian cells (12). Consequently, Myc expression in normal cells is tightly regulated. Following damage in a regenerative tissue, the release of mitogens stimulates a transient increase in the short-lived Myc protein followed by a transient proliferative response (13). In contrast, Myc transcriptional programmes decrease during postnatal cardiac maturation and fail to reactivate post-infarction (14). Moreover, even when Myc expression is driven ectopically in the adult mammalian heart, it is unable to activate transcription of many target genes and cardiomyocytes remain almost entirely resistant to proliferation (11, 15). We previously established that Myc-driven transcription, and consequently cell proliferation, are critically dependent on the level of Cyclin T1 in cardiomyocytes (11).

Cyclin T1 strongly associates with CDK9 to form the positive transcription elongation factor b (P-TEFb) (16) that phosphorylates paused RNA PolII and the elongation factors DSIF and NELF, leading to productive transcriptional elongation (16, 17). Both components of P-TEFb, Cyclin T1 and CDK9 are tightly regulated by various transcriptional and post-transcriptional mechanisms (18, 19). P-TEFb is also dynamically controlled by an association with an inactivation complex (7SK snRNA, Larp7, MEPCE, and HEXIM) (20, 21). Unlike other cyclin-dependent kinases, CDK9 protein stability is not cell cycle dependent (22), but is primarily determined by binding to Cyclin T1 (23, 24). The level of the Cyclin T1 protein is thus the key factor in controlling the amount of the P-TEFb complex in a cell (23, 24). During postnatal cardiac maturation in mice the level of P-TEFb and phosphorylated RNA PolII steadily decline (11). Transgenic overexpression of Cyclin T1 in the heart leads to increased levels of both CDK9 and phosphorylated RNA PolII and, when expressed throughout development, leads to cardiac hypertrophy (11, 25, 26).

The RAS proteins (K-RAS, N-RAS, and H-RAS in humans) are small GTPases that function as molecular switches, cycling between their “off” GDP-bound and “on” GTP-bound states in response to mitogenic signalling (27). Depending on the cellular context, Ras can activate several downstream pathways that regulate protein synthesis, cell growth, survival, and cell motility (28), in the heart, Ras activation leads to reversible cardiac hypertrophy (29, 30). Hypertrophy, be it transgenically induced by constitutively active RAS or secondary to pressure overload or other hypertrophic stimuli, is accompanied by an increase in total RNA content. This is a result of increased P-TEFb activity and phosphorylation of RNA Pol II that increases total RNA and protein synthesis (25, 31). Using a genetically modified mouse model that combines the elevated expression of HRas*^G^*^12^*^V^* and an ectopically switchable Myc allele (MycER*^T^*^2^), we show that expression of constitutively active HRas*^G^*^12^*^V^* in the heart leads to increased P-TEFb levels that, when combined with constitutive Myc activity, drives signs of cardiomyocytes proliferation *in vivo*.

## Materials and methods

### Mice

All animals were kept under SPF conditions. Mice were maintained on regular diet in a pathogen-free facility on a 12 h light/dark cycle with continuous access to food and water. All mice were euthanised under the schedule 1 method of cervical dislocation. Mouse strain *Tg(Myh6-tTA)6Smbf/J* was obtained from the Jackson Laboratory, and mouse strain *Tg(tetO-HRAS)65Lc/Nci* was obtained from the NCI Mouse Repository. Mouse strain *R26^CAG–c–MycER^* was produced in house. 1 mg of (Z)-4-hydroxytamoxifen (4-OHT; Sigma, H7904) was i.p injected into adult mice in 10% ethanol and vegetable oil (5 mg/ml), and tissues were collected 4 h post injection. 1 mg of tamoxifen (Sigma, T5648) was i.p injected into adult mice in 10% ethanol and vegetable oil (10 mg/ml) twice over a 24-h period and tissues collected at 24 h post initial i.p. injection. Animals requiring doxycycline treatment were supplied with drinking water containing doxycycline hyclate 100 mg/L (Sigma D9891) in water containing 3% sucrose to increase palatability, this was replenished two times per week in light-protected bottles. Ear biopsies were collected from 2 to 5 weeks old mice and genotyped by PCR with the following oligonucleotide primers: for *Rosa26CAG*; Universal forward: 5′-CTCTGCTGCCTCCTGGCTTCT-3′ Wild-type reverse: 5′-CGAGGCGGATCACAAGCAATA-3′ and CAG reverse: 5′-TCAATGGGCGGGGGTCGTT-3′. Primers for the TetO-HRas allele were; H003: 5′-TGAAAGTCGAGCTCGGTA-3′ and H004: 5′-CCCGGTGTCTTCTATGGA-3′. Primers for the Myh6-tTA allele were; oIMR8746: 5′-CGCTGTGGG GCATTTTACTTTAG-3′ and oIMR8747: 5′-CATGTCCAGAT CGAAATCGTC-3′.

### Immunoblotting

Snap-frozen animal tissues were ground on liquid nitrogen and proteins extracted in buffer containing 1% SDS, 50 mM Tris pH 6.8, and 10% glycerol on ice for 10 min. Lysates were boiled for 10 min, followed by sonication for 15 min at room temperature. Total protein (50 μg) was electrophoresed on an SDS–PAGE gel and transferred onto immobilon-P (Millipore) membranes. These were then blocked in 5% non-fat milk and incubated with primary antibodies overnight at 4°C. Secondary antibodies were applied for 1 h followed by chemiluminescent visualisation (Thermo Scientific, 32106 or Millipore, WBKLS0500). Immunoblots were either developed on Fuji RX X-ray film and scanned on an Epson Perfection V500 Photo flatbed scanner or visualisation was performed on LiCOR Odyssey Fc. Protein quantifications were performed on the LiCOR Odyssey Fc.

Primary antibodies: GAPDH (D16H11) XP^®^ (Cell Signaling Technology, 5174, used at 1:5,000), phospho-Rpb1 CTD (Ser2; E1Z3G; Cell Signaling Technology, 13499, used at 1:2,500), CDK9 (C12F7; Cell Signaling Technology, 2316, used at 1:1,000), Cyclin T1 (D1B6G; Cell Signaling Technology, 81464, used at 1:1,000), p-ERK is phospho-p44/42 MAPK (Thr202/Tyr204, Cell Signaling Technology, 9101, used at 1:500), anti-rabbit IgG HRP (Sigma, A0545, used at 1:10,000). Sample loading was normalised for equal protein content. Expression of GAPDH is included as a confirmation of efficiency of protein isolation and comparable loading between individual tissue samples.

### Immunohistochemistry

Immunohistochemistry was performed on formalin-fixed paraffin-embedded 4 μm sections. Sections were de-paraffinized and rehydrated, followed by antigen retrieval by boiling in 10 mM citrate buffer (pH 6.0) for 10 min. Endogenous peroxidase activity was blocked with 0.3% hydrogen peroxide for 30 min. Sections were then treated with rabbit VECTASTAIN Elite ABC horseradish peroxidase kit (Vector Laboratories, PK-6101) following the manufacturer’s protocols. Sections were blocked in normal goat serum for 20 min, followed by 1 h incubation in the primary antibody at room temperature. Sections were then washed three times in PBST and incubated for 1h in secondary antibody, sections were then washed again in PBST and then incubated in ABC complex for 30 min. Sections were developed in DAB (3,3’-diaminobenzidine) for 5 min, counterstained in haematoxylin, dehydrated, and mounted in DPX. Staining was imaged on a Zeiss Axio Imager using the Zeiss ZEN software using the AutoLive setting and interactive white balance.

Primary antibodies: p-ERK is phospho-p44/42 MAPK (Thr202/Tyr204, Cell Signalling Technology, 9101, used at 1:500), and anti-Cyclin T1 (AbCam, ab238940, use at 1:500).

### Immunofluorescence

Sections were pre-processed followed by antigen retrieval in the same way as for immunohistochemistry. Sections were blocked in 2.5% goat serum, and 1% BSA in PBST for 20 mins at room temperature. Primary antibody, made up of blocking buffer, was added for 1 h at room temperature, followed by three 5 min PBST washes, and secondary antibodies were added for 1 h at room temperature. Nuclei were visualised using Hoechst (Sigma, 861405) and sections mounted in ProLong™ Gold Antifade Mountant (Thermo Fisher, P36930). Antibodies: anti-phospho-Histone H3 (Ser10; Merck Millipore, 06-570, use at 1:500), cardiac troponin T (13-11; Thermo Fisher, MA5-12969, use at 1:100) and (CT3, Santa Cruz Biotechnology, sc-20025, use at 1:50), anti-Aurora B antibody (Abcam, ab2254, use at 1:200), anti-Mklp1 (Abcam, ab174304, use at 1:400), anti-CD206 (R&D Systems, AF2535, use at 1:100), anti-PCM1 (Sigma-Aldrich, HPA023370, use at 1:100), anti-Ki67 (SolA15; Thermo Fisher, use at 1:100) and (Atlas Antibodies, HPA023374, use at 1:100), Alexa Fluor 555 goat anti-rabbit IgG (H+L; Life Technologies, A21428), Alexa Fluor 555 goat anti-mouse IgG (H+L; Life Technologies, A21422), Alexa Fluor 555 goat anti-rat IgG (H+L; Life Technologies, A21434), Alexa Fluor 488 goat anti-rabbit IgG (H+L; Life Technologies, A11008), Alexa Fluor 488 goat anti-rat IgG (H+L; Life Technologies, A11006), Alexa Fluor 350 goat anti-mouse (H+L; Life Technologies A11045), wheat germ agglutinin, Alexa Fluor™ 488 conjugate (Thermo Fisher, W11261). TUNEL staining we performed following the manufactures instructions using the ApopTag^®^ Fluorescein *In Situ* Apoptosis Detection Kit (S7110 Sigma-Aldrich). Staining was imaged on a Zeiss Axio Imager using the Zeiss ZEN software using the AutoLive setting or Leica Stellaris 5 confocal microscope. Quantification was performed by counting the number of positive cardiomyocytes for at least five images per organ/mouse, the mean of five raw counts was calculated and is represented by each data point per graph. Cardiomyocyte cell areas were quantified by encircling individual cardiomyocytes stained with cardiac troponin and wheat germ agglutinin.

### Quantitative RT-PCR

Total RNA was isolated using TRIzol Reagent (Thermo Fisher, 15596-018) following manufacturer’s instructions. cDNA was synthesised from 1 μg of RNA using the High-Capacity cDNA Reverse Transcription Kit with RNase Inhibitor (Thermo Fisher, 4374966) following manufacturer’s instructions. qRT-PCR reactions were performed on an Applied Biosystems QuantStudio 5 Real-Time PCR System using Fast SYBR Green Master Mix (Thermo Fisher, 4385612), following manufacturer’s instructions. Primers: Ccnd1; forward 5′- GCGTACCCTGACACCAATCTC-3′, reverse 5′-CTCCTCTTCGCACTTCTGCTC-3. Cdk4; forward 5′-ATGGCTGCCACTCGATATGAA-3′, reverse 5′-TC CTCCATTAGGAACTCTCACAC-3′. Cdk1; forward 5′- AGAA GGTACTTACGGTGTGGT-3′, reverse 5′-GAGAGATTTCCC GAATTGCAGT-3′. Ccnb1; forward 5′-AAGGTGCCTGT GTGTGAACC-3′, reverse 5′-GTCAGCCCCATCATCTGCG-3′. Cad; forward 5′- CTGCCCGGATTGATTGATGTC-3′, reverse 5′-GGTATTAGGCATAGCACAAACCA-3′. Pinx1; forward 5′-AGCAAGGAGCCACAGAACATA-3′, reverse 5′-GGTGAGCAATCCAGTTGTCTT-3′. Bzw2; forward 5′-AGC GACTGTCTCAGGAATGC-3′, reverse 5′-CTGTTTCCGGAA GGTCGTT-3′. Polr3d; forward 5′-AAAAGCGTGAACGGGAC AGG-3′, reverse 5′-AATGGGACTGGATCACTTCCG-3′. St6galnac4; forward 5′-TGGTCTACGGGGATGGTCA-3′, reverse 5′-CTGCTCATGCAAACGGTACAT-3′. Actin; forward 5′-GACGATATCGCTGCGCTGGT-3′, reverse 5′-CCACGATG GAGGGGAATA-3′. Gapdh; forward 5′-AGGTCGGTGTGA ACGGATTTG-3′, reverse 5′-TGTAGACCATGTAGTTGAGG TCA-3′. Fosl1; forward 5′-ATGTACCGAGACTACGGGGAA-3′, reverse 5′-CTGCTGCTGTCGATGCTTG-3′. Egr3; forward 5′-CCGGTGACCATGAGCAGTTT-3′, reverse 5′-TAATGGG CTACCGAGTCGCT-3′.

### Bulk RNA sequencing

Total RNA was purified using PureLink RNA Mini Kit (Thermo Scientific). On-column DNA digestion was performed using PureLink DNase (Thermo Scientific). 1 μg of purified RNA was treated with Ribozero rRNA removal kit (Illumina). RNA quality and removal of rRNA were checked with the Agilent 2100 Bioanalyser (Agilent Technologies). Libraries for RNA-seq were then prepared with the NEBNext^®^ Ultra™ II DNA Library Prep Kit for Illumina (NEB) following the manufacturer’s instructions starting from the RNA fragmentation step.

### Bulk RNA sequencing data analysis

Sequence reads were adapter trimmed using Cutadapt (32) (version 1.11) and aligned using BWA aln for short reads (version 2.5.2a) to the GRCm38 (mm10) assembly with the gene, transcript, and exon features of the Ensembl (release 70) gene model. Expression was estimated using RSEM (33) (version 1.2.30). Transcripts with zero read counts across all samples were removed prior to analysis. Normalisation of RSEM expected read counts was performed by dividing by million reads mapped to generate counts per million (CPM), followed by the trimmed mean of M-values (TMM) method from the edgeR package (34). Differential expression analysis was performed using edgeR.

### RNA sequencing comparison to myocardial infarction reversion network

RNA-seq files from E-MTAB-7595, E-MTAB-7636, and GSE95755 were combined into a single count matrix. Before remapping, poor quality sequences (<30 phred score) and adapter sequences were trimmed with Trimmomatic (35). Reads were mapped with STAR (36) to the reference mouse genome sequence GCRm38.p4/mm10 using default settings. The combined count matrix was generated using HTSeq-count (37) on union mode. Differential expression analysis was performed with EdgeR(v3.20.8) (34) using the glmLRT function. Differential expression comparisons were considered significant if FDR *p* < 0.05. Gene ontological analysis was performed using DAVID and Heatmaps were assembled using GENE-E (Broad Institute).

### Statistical analysis

Bioinformatic and statistical analyses were performed using R with Bioconductor packages and comEpiTools packages (38, 39). Gene lists from RNA and ChIP sequencing were analysed in Enrichr or GSEA/MSigDB^1^ (40). Statistical analyses for IHC and q-RT-PCR were performed using GraphPad Prism v9.0d (GraphPad Software, Inc., San Diego, CA, USA) as indicated with *p* ≤ 0.05 considered to be statistically significant. Venn diagrams were drawn in the Lucidchart.^2^ Heatmaps were drawn in R and Morpheus.^3^

## Results

### Cardiac *HRas*^G12V^ expression leads to hypertrophy and amplified P-TEFb expression

**FIGURE 1:**
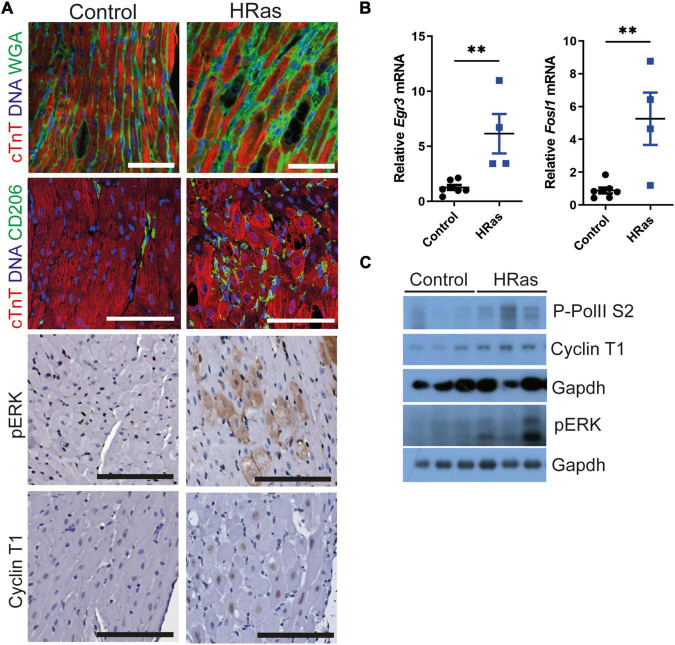
Ectopic cardiomyocyte HRas^G12V^ results in increased Cyclin T1 expression. **(A)** Top- Immunofluorescent staining of cardiac troponin (red) and wheat germ agglutinin or CD206 (green). middle- immunohistochemical analysis of pERK, bottom- immunohistochemical analysis of Cyclin T1 in hearts from wild type (Control) and *TetO-HRas; Myh6-tTA; R26*^+/+^(*HRas*) mouse hearts 4 weeks post withdrawal of doxycycline. Bars are 100 μm. Representative images based on analysis of 5 images per mouse. **(B)** Quantitative RT-PCR analysis of control (*R26*^+/+^*; TetO-HRas, R26*^+/+^*; Myh6-tTA* or *R26*^+/+^, *n* = 7) and *TetO-HRas; Myh6-tTA; R26*^+/+^(*R26*^+/+^*;HRas, n* = 4) mouse hearts 4 weeks post withdrawal of doxycycline. Expression is normalised to *Actin* and *Gapdh* and relative to the respective control. Replicate samples are derived from independent mice. Error bars show s.e.m. Two Way ANOVA with multiple comparisons test. **p < 0.01. **(C)** Immunoblot analysis of the C-terminal domain of phosphorylated RNA Polymerase II [p-Rpb1(S2)], Cyclin T1 and pERK protein expression in hearts isolated from control (*R26*^+/+^*; TetO-HRas, R26*^+/+^*; Myh6-tTA* or *R26*^+/+^) and *TetO-HRas; Myh6-tTA; R26*^+/+^(*HRas*) mice 4 weeks post withdrawal of doxycycline. Expression of Gapdh is included as a loading control, for completeness, multiple Gapdh blots represent different gels (**top** – Cyclin T1 and P-RNA PolII S2, **bottom** – pERK). Replicate samples are derived from independent mice.

We employed the cardiac-specific, doxycycline-controlled transgenic line *TetO-HRas* [*Tg(tetO-HRAS)65Lc/Nci]; Myh6tTA [Tg(Myh6-tTA)6Smbf/Jm*] (30) to overexpress HRas^G12V^ specifically in cardiomyocytes. Mice were generated and removed from doxycycline treatment at 4 weeks of age and the subsequent expression of constitutively active HRas^G12V^ led to an increase in hypertrophic cardiomyocytes (∼10%, Supplementary Figure 1A) interspersed with CD206 positive macrophages and cardiomyocytes of normal volume (Figure 1A), as previously described (30). Hypertrophic cardiomyocytes displayed elevated pERK immunohistochemical staining (Figure 1A) and qRT-PCR analysis confirmed increased expression of the ERK-AP1 target genes Egr3 and Fos1L (Figure 1B), confirming enhanced HRas signalling. Despite the heterogeneity of the model, whole hearts expressing HRas^G12V^ displayed a moderate increase in Cyclin T1 and Phosphorylated RNA Pol II (Figures 1A,C and Supplementary Figure 1B).

### HRas^G12V^ enables Myc-driven transcription in cardiomyocytes

**FIGURE 2:**
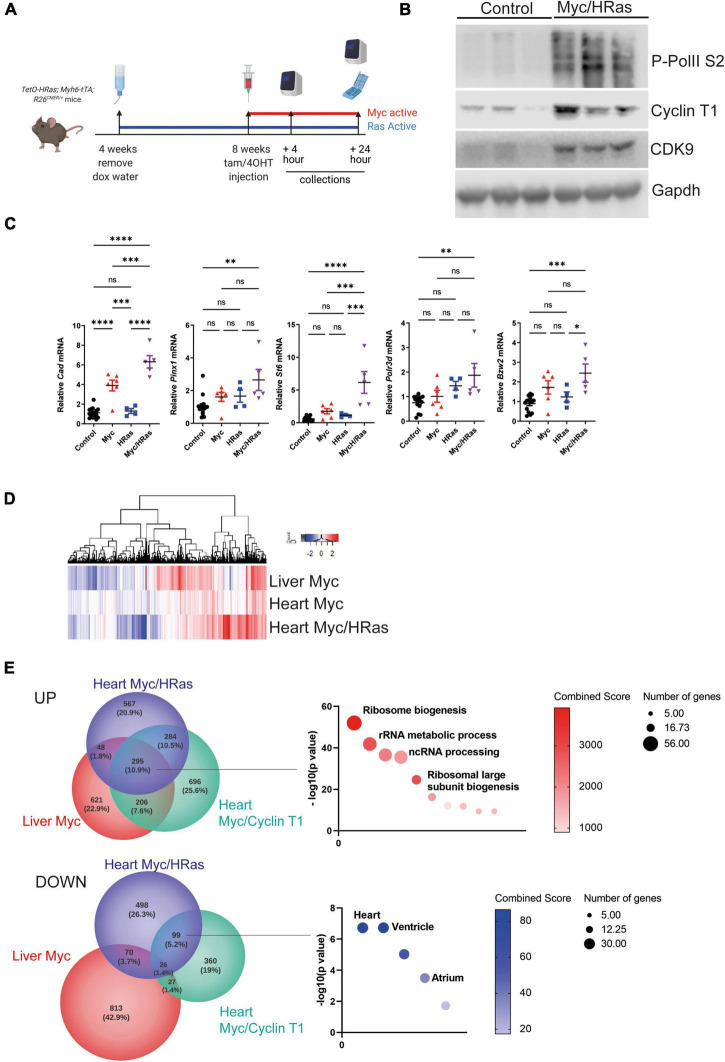
Cardiomyocyte HRas^G12V^ expression enables Myc-driven transcription. **(A)** Scheme of experimental work. **(B)** Immunoblot analysis of the C-terminal domain of phosphorylated RNA Polymerase II [p-Rpb1(S2)], Cyclin T1 and CDK9 protein expression in hearts isolated from control (*R26*^+/+^*; TetO-HRas, R26*^+/+^*; Myh6-tTA* or *R26*^+/+^) and *TetO-HRas; Myh6-tTA; R26* ^CMER/+^(HRas/Myc) mice 4 weeks post withdrawal of doxycycline and at 24 h post administration of 4-OHT. Expression of Gapdh is included as a loading control. Replicate samples are derived from independent mice. **(C)** Quantitative RT-PCR analysis of *Cad, Bzw2, Pinx1, Polr3d, St6*, and *Cdc25a* in hearts from control (*R26*^+/+^*; TetO-HRas, R26*^+/+^*; Myh6-tTA* or *R26*^+/+^, *n* ≥ 14), *TetO-HRas; Myh6-tTA; R26*^+/+^(*HRas, n* = *4*), *R26*^CMER/+^ (*Myc*, *n* = *6*) and *TetO-HRas; Myh6-tTA; R26* ^CMER/+^ (*MycHRas, n* = *5*) mice 4 weeks post withdrawal of doxycycline and 4 h post-administration of 4-OHT. Expression is normalised to *Actin* and *Gapdh* and relative to the respective control. Error bars show s.e.m. Two Way ANOVA with multiple comparisons test; control vs *R26*^CMER/+^: p*** = 0.001 (*Cad*), control vs *TetO-HRas; Myh6-tTA; R26* ^CMER/+^: p** = 0.01 (*Bzw2, Pinx1, Polr3d, Cdc25a*) p*** = 0.001 (*St6*). Replicate samples are derived from independent mice. **(D)** Heat map showing the union of DEGs called in adult liver and adult heart. Shown are mRNA expression fold changes (Log2) upon MycER^T2^ activation relative to wild-type, as determined by RNA sequencing of *R26*^CMER/+^ (*n* ≥ 3) mice in comparison to wild-type (*R26*^+/+^, *n* ≥ 3) and *TetO-HRas; Myh6-tTA; R26* ^CMER/+^ mice (*Myc/HRas, n* = 3) in comparison to *TetO-HRas; Myh6-tTA; R26*^+/+^ mice (*n* = 3) at 4 h post administration of 4-OHT. **(E)** Venn diagrams of overlap in the number of genes showing an increase (top) and decrease (bottom) in expression in response to supraphysiological Myc expression. The most significant GO Biological Process and ASCHS4 gene lists that overlap are shown. Comparisons included the liver of *R26*^CMER/+^ (Myc liver, *n* = 3) compared to wild type. The adult mouse heart was isolated 4 weeks post systemic infection with an adeno associated virus encoding *Ccnt1* (*AAV9-Ccnt1*) compared to control and heart from *TetO-HRas; Myh6-tTA; R26* ^CMER/+^ (*n* = 3) 4 weeks after HRas^G12D^ expression and 4 h post Myc activation compared to control, as determined by RNA sequencing (FDR < 0.05 and abs(log2FC) > 0.5).

To test the hypothesis that persistent HRas^G12V^ expression renders heart tissue Myc responsive, *TetO-HRas; Myh6tTA* mice were crossed to the *R26*^CMER/+^ (*R26*^CAG–c–MycERT2^) allele to generate *TetO-HRas; Myh6-tTA; R26*^CMER/+^ mice. In these mice, the MycER^T2^ fusion protein is constitutively expressed from a CAG-enhanced *Rosa26* promoter. MycER^T2^ is inert in the absence of a ligand but rapidly activated following administration of the ER^T2^ ligand 4OHT (4-hydroxytamoxifen), a primary metabolite of tamoxifen. In these mice, HRas^G12V^ and MycER^T2^ proteins can be independently activated by the withdrawal of doxycycline or administration of 4OHT (or tamoxifen), respectively (Figure 2A). Doxycycline was withdrawn from *TetO-HRas; Myh6-tTA; R26*^CMER/+^ animals at weaning to induce expression of HRas^G12V^ and, once mice reached 8 weeks of age, MycER^T2^ was activated by injection of 4OHT for 4 h. We confirmed the elevated expression of Cyclin T1, CDK9, and phosphorylated RNA Pol II in the heart tissue of these animals compared to controls (Figure 2B and Supplementary Figure 1C).

We have previously determined that Myc-driven transcription is limited by the low level of endogenous P-TEFb within the adult heart. To establish if an HRas^G12V^-driven increase in P-TEFb levels could enhance Myc-driven transcription, we quantified the expression of a panel of Myc target genes post MycER^T2^ activation. Cad, a Myc target gene previously shown to be transcribed following MycER^T2^ activation in all tissues, including the heart, showed increased expression in the heart tissue of both Myc activated (*R26*^CMER/^) and HRas^G12V^/Myc activated (*TetO-HRas; Myh6-tTA; R26^CMER/+^)* tissues, confirming the functionality of the MycER^T2^ fusion in both conditions (Figure 2C). We then determined the expression of a panel of Myc target genes that we have previously observed as unchanged in the adult heart in response to MycER^T2^ activation alone. The co-expression of HRas^G12V^ sensitised the heart tissue to Myc-dependent expression of these targets over controls (Figure 2C). We also compared changes in global gene expression in heart of HRas^G12V^ (*TetO-HRas; Myh6-tTA; R26*^+/+^) mice and Myc/HRas^G12V^ (*TetO-HRas; Myh6-tTA; R26* ^CMER/+^) mice 4 h after MycER^T2^ activation. We have previously observed marked induction of a Myc-driven transcriptional programme in the liver of MycER^T2^ expressing (*R26 ^CMER/+^)* mice in the absence of co-expressed HRas^G12V^ (11) (Figure 2D). In contrast to the weak transcriptional response elicited by MycER^T2^ activation alone, in the presence of HRas^G12V^, MycER^T2^ resulted in a marked transcriptional response (Figure 2D). MycER^T2^ and HRas^G12V^ expressing hearts displayed 1198 up-regulated DEGs and 693 down regulated DEGs (Supplementary Table 1) in comparison to HRas^G12V^ expression alone. Myc target gene identities overlapped with genes induced by MycER^T2^ in the adult liver, and in the adult heart in the presence of elevated Cyclin T1 expression (AAV9-driven cardiomyocyte-specific) (11) (Figure 2E). These genes are Myc targets, involved in direct Myc-regulated processes such as ribosomal biogenesis and RNA metabolic processes (Figure 2E, Supplementary Figure 1D, and Supplementary Table 2). In contrast, there was little overlap between downregulated genes observed following MycER^T2^ and HRas^G12V^ activation in the heart and genes downregulated by MycER^T2^ alone in the liver, suggesting a level of tissue specificity for Myc-dependent transcriptional repression. Consistent with this, genes downregulated in response to HRas^G12V^ and MycER^T2^ overlapped with those downregulated in the MycER^T2^ and Cyclin T1 expressing heart – these genes were characteristic of a homeostatic heart transcriptional programme, such as HCN4, MYOT (Figure 2E and Supplementary Table 3).

**FIGURE 3:**
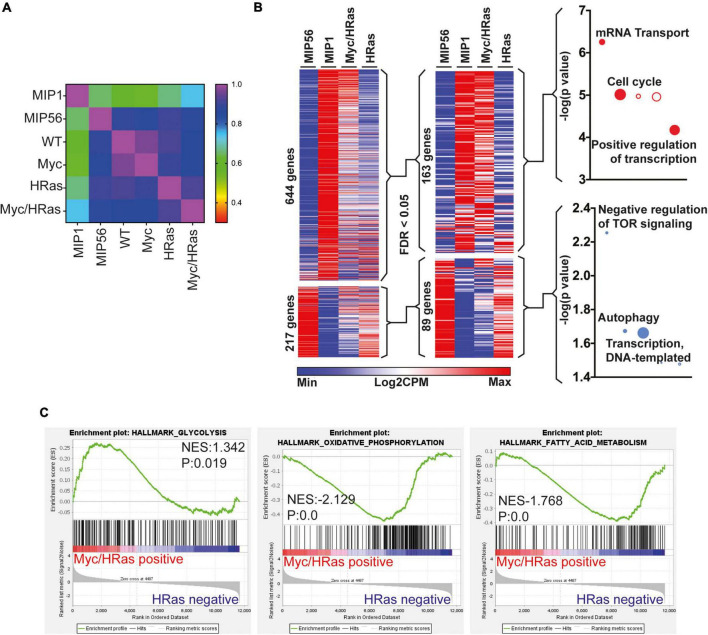
Cardiac MycER^T2^ and HRas^G12V^ expression result in signalling characteristic of neonatal cardiomyocytes. **(A)** Pearson correlative analysis of regenerative network genes (described in ^14^) expressed in day 1 myocardial infarcted heart myocytes (MIP1), adult day 56 myocardial infarcted heart myocytes (MIP56), adult *R26*^+/+^ (WT) hearts, adult *R26* ^CMER/+^ (Myc) hearts, adult *TetO-HRas; Myh6-tTA; R26*^+/+^ (*HRas*) and adult *TetO-HRas; Myh6-tTA; R26* ^CMER/+^ (*Myc/HRas*) RNA-seq datasets. **(B)** Heat map depicts gene expression of the regeneration network genes (described in ^14^) from RNA-seq of *TetO-HRas; Myh6-tTA; R26*^+/+^ (*HRas, n* = 3) and *TetO-HRas; Myh6-tTA; R26* ^CMER/+^ P60 adult hearts (*Myc/HRas, n* = 3). MIP1.Myo and MIP56.Myo denotes neonatal (P1) or adult (P56) cardiomyocytes isolated from myocardial infarction-operated hearts (*n* = 4). Regeneration network genes were filtered for genes differentially expressed (DEGs) between *TetO-HRas; Myh6-tTA; R26*^+/+^ (*HRas*) and *TetO-HRas; Myh6-tTA; R26* ^CMER/+^ (*Myc/HRas*) hearts (FDR < 0.05). **(C)** Gene set enrichment analysis of glycolysis, oxidative phosphorylation, and fatty acid metabolism gene lists in comparison to differential gene expression observed between *TetO-HRas; Myh6-tTA; R26* ^CMER/+^ mice (*Myc/HRas, n* = 3) and *TetO-HRas; Myh6-tTA; R26*^+/+^ mice (*n* = 3) at 4 h post administration of 4-OHT.

To determine if the transcriptional changes observed in response to combined activation of MycER^T2^ and HRas^G12V^ in the adult heart were similar to that observed in a regenerating neonatal heart, we analysed previous data to compare the transcriptional profiles of cardiomyocytes isolated from surgically infarcted hearts at different stages of development (14). Our unbiased comparison revealed that the P1 “neonatal regeneration gene expression signature” correlated most closely with the expression changes within the *TetO-HRas; Myh6-tTA; R26*^CMER/+^ hearts (Figure 3A). Upregulated genes that overlapped with those also elevated in the “neonatal regeneration gene expression signature” enriched for GO terms including cell cycle control (Figure 3B). Interestingly, downregulated genes that overlapped with those also reduced in the “neonatal regeneration gene expression signature” contained genes involved in the negative regulation of mTOR signalling and we have previously observed a broad reprogramming of metabolism with a transition from neonatal to adult cardiac maturation (41). Furthermore, when regulated metabolic genes from our analysis are overlaid on the KEGG metabolic pathways *TetO-HRas; Myh6-tTA; R26*^CMER/+^ hearts show broad reversion and reprogramming to neonatal metabolic pathways (Supplementary Figure 2A). Consistent with this reversion, gene set enrichment analysis (GSEA) indicated MycER^T2^ and HRas^G12V^ expressing hearts display enrichment of genes involved in glycolysis, characteristic of neonatal cardiomyocyte metabolism, and a reduction in genes involved with oxidative phosphorylation and fatty acid metabolism characteristic of adult cardiomyocyte metabolism (Figure 3C).

### Combined HRas and Myc expression drives proliferation of cardiomyocytes *in vivo*

**FIGURE 4:**
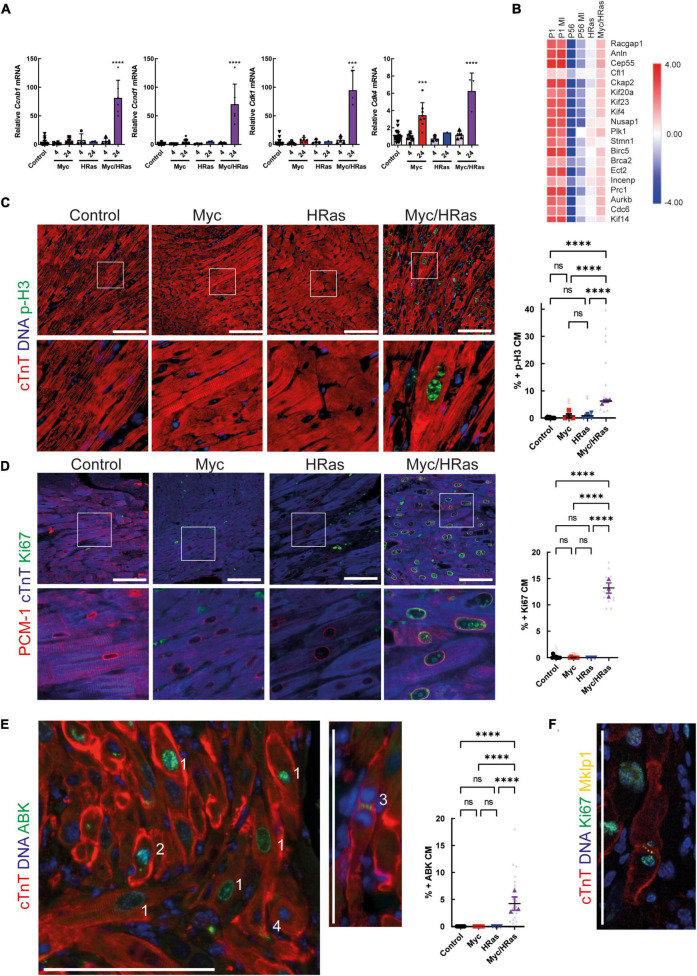
MycER^T2^ and HRas^G12V^ expression drive cardiomyocyte proliferation. **(A)** Quantitative RT-PCR analysis of *Cyclin D1, Cdk4, Cyclin B1*, and *Cdk1* in hearts of control (*R26*^+/+^*; TetO-HRas, R26*^+/+^*; Myh6-tTA* or *R26*^+/+^, *n* ≥ 24), *TetO-HRas; Myh6-tTA; R26*^+/+^(*R26*^+/+^*;HRas, n* ≥ 5), *R26*^CMER/+^ (*n* ≥ *14*) and *TetO-HRas; Myh6-tTA; R26* ^CMER/+^ (*R26* ^CMER/+^*;HRas, n* = *8*) mice 4 weeks post withdrawal of doxycycline and 4 or 24 h post administration of 4-OHT. Expression is normalised to *Actin* and *Gapdh* and relative to the respective control. Error bars show s.e.m. One Way ANOVA with multiple comparisons test; control *vs* 24 h *R26*^CMER/+^: *p* = 0.001*** (*Cdk4*), control *vs* 24 h *TetO-HRas; Myh6-tTA; R26* ^CMER/+^: p**** = 0.0001 (*Cyclin D1, Cdk4, Cyclin B1, Cdk1*). Replicate samples are derived from independent mice. **(B)** Heat map depicts gene expression (LogCPM) of cytokinesis genes in the regeneration network (described in ^14^) from RNA-seq of adult *TetO-HRas; Myh6-tTA; R26*^+/+^ (*HRas, n* = 3) and adult *TetO-HRas; Myh6-tTA; R26* ^CMER/+^ hearts (*Myc/HRas, n* = 3), neonatal (P1) or adult (P56) cardiomyocytes isolated from sham and myocardial infarction-operated (MI) hearts (*n* = 4). **(C)** Left- immunofluorescent staining of heart from control, *TetO-HRas; Myh6-tTA; R26*^+/+^ (*HRas*), *R26*^CMER/+^ (Myc) and *TetO-HRas; Myh6-tTA; R26* ^CMER/+^ (Myc/*HRas*) mice 4 weeks post-withdrawal of doxycycline and 24 h post administration of tamoxifen. Cardiac troponin (red) and p-H3 positive mitotic nuclei (green). Representative images based on analysis of at least 3 independent mice. Right- quantification of percent p-H3 positive cardiomyocyte in hearts isolated from control (*R26*^+/+^*; TetO-HRas, R26*^+/+^*; Myh6-tTA* or *R26*^+/+^, black, *n* = 8), *TetO-HRas; Myh6-tTA; R26*^+/+^ (*HRas*, red, *n* = 3), *R26*^CMER/+^ (Myc, blue, *n* = 4) and *TetO-HRas; Myh6-tTA; R26* ^CMER/+^ (*Myc/HRas*, purple, *n* = 3) mice 4 weeks post withdrawal of doxycycline and 24 h post administration of tamoxifen. Bold symbols = average per mouse heart, small faint symbols = each individual field quantified. Bars are 100 μm. Representative images based on analysis of 5 images per mouse; error bars show s.e.m. One Way ANOVA with multiple comparisons test: *p* < 0.0001****. **(D)** Left- Immunofluorescent staining of PCM-1 (red) and Ki67 (green) in the heart from control, *TetO-HRas; Myh6-tTA; R26*^+/+^ (*HRas*), *R26*^CMER/+^ (Myc) and *TetO-HRas; Myh6-tTA; R26* ^CMER/+^ (Myc/*HRas*) mice 4 weeks post withdrawal of doxycycline and 24 h post administration of tamoxifen. Right Myc/HRas image shows relocation of PCM1 and cardiac troponin signal in mitotic cardiomyocytes. Right- quantification of percent Ki67 positive cardiomyocytes in hearts isolated from control (*R26*^+/+^*; TetO-HRas, R26*^+/+^*; Myh6-tTA* or *R26*^+/+^, black, *n* = 10), *TetO-HRas; Myh6-tTA; R26*^+/+^ (*HRas*, red, *n* = 3), *R26*^CMER/+^ (Myc, blue, *n* = 7) and *TetO-HRas; Myh6-tTA; R26* ^CMER/+^ (*Myc/HRas*, purple, *n* = 3) mice 4 weeks post withdrawal of doxycycline and 24 h post administration of tamoxifen. Bold symbols = average per mouse heart, small faint symbols = each individual field quantified. Bars are 100 μm. Representative images based on analysis of at least 3 independent mice. One Way ANOVA with multiple comparisons test: *p* < 0.0001****. **(E)** Left- Immunofluorescent staining of cardiac troponin (red) and Aurora B positive mitotic nuclei (green) in the heart of *TetO-HRas; Myh6-tTA; R26* ^CMER/+^ (Myc/*HRas*) mice 24 h post administration of tamoxifen. Numbers indicate differences in Aurora B localisation throughout the cell cycle. 1- Prophase and Prometaphase, 2- Metaphase, 3- Late anaphase/early telophase, 4 – centrally located mid-body. Right- quantification of percent ABK positive cardiomyocytes in hearts isolated from control (*R26*^+/+^*; TetO-HRas, R26*^+/+^*; Myh6-tTA* or *R26*^+/+^, black, *n* = 8), *TetO-HRas; Myh6-tTA; R26*^+/+^ (*HRas*, red, *n* = 4), *R26*^CMER/+^ (Myc, blue, *n* = 7) and *TetO-HRas; Myh6-tTA; R26* ^CMER/+^ (*Myc/HRas*, purple, *n* = 3) mice 4 weeks post withdrawal of doxycycline and 24 h post administration of tamoxifen. Bold symbols = average per mouse heart, small faint symbols = each individual field quantified. Bars are 100 μm. Representative images based on analysis of at least 3 independent mice. Kruskal–Wallis with Dunn’s multiple comparisons: *p* < 0.01**, *p* < 0.001***. **(F)** Immunofluorescent staining of cardiac troponin (red), Ki67 (green) and Mklp1 (orange) in the heart of *TetO-HRas; Myh6-tTA; R26* ^CMER/+^ (Myc/*HRas*) mice 24 h post administration of tamoxifen. Bars = 100 μm.

To determine if this Myc-dependent “regenerative transcriptional response” results in productive proliferation, MycER^T2^/HRas^G12V^ expressing mice (*TetO-HRas; Myh6-tTA; R26*^CMER/+^) or mice expressing either HRas^G12V^ or MycER^T2^ alone were administered tamoxifen via intraperitoneal injection at 8 weeks of age to activate MycER^T2^ and hearts collected after 24 h. Transcriptional analysis indicated that activation of MycER^T2^ in the presence of HRas^G12V^ induced robust expression of multiple cell cycle genes (*Cdk4*, *Ccnd1*, *Cdk1*, *Ccnb1*) that were not induced by HRas^G12V^ or Myc activation alone (except *Cdk4* which is a known Myc target, Figure 4A). Furthermore, markers of cytokinesis were upregulated in Myc and HRas^G12V^ expressing hearts (Figure 4B) and GSEA indicated that these hearts displayed significant enrichment for genes involved in cell cycle-related pathways such as E2F targets and G2/M checkpoints (Supplementary Figure 2B). These transcriptional events also underpinned significant markers of cell-cycle progression indicated by Ki67 (general cell cycle) and p-H3 (mitotic) positive cardiomyocytes specifically marked by PCM1 and cardiac troponin T, respectively (Figures 4C,D). In addition, Aurora B kinase displayed staining representative of cell cycle progression (prophase, metaphase, late anaphase/telophase, and mid body localisation) combined with features of disassembled sarcomeres that become marginalised to the cell periphery (4, 42) (Figure 4E and Supplementary Figure 3). Furthermore, Mklp1 expression was observed in cardiomyocytes at the cleavage furrow (Figure 4F).

### Combined HRas^G12V^ and MycER^T2^ expression leads to apoptosis of cardiomyocytes

**FIGURE 5:**
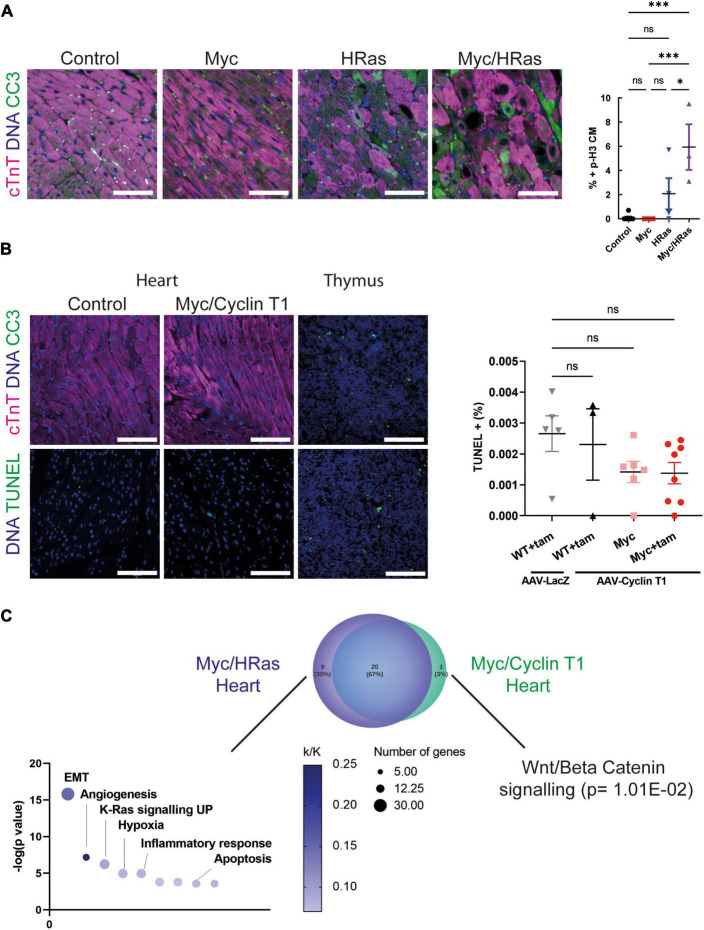
Cardiac HRas^G12V^ and MycER^T2^ expression results in cardiomyocyte apoptosis. **(A)** Left- immunofluorescent staining of cleaved caspase 3 (green) and cardiac troponin (pink) in heart from control, *TetO-HRas; Myh6-tTA; R26*^+/+^ (*HRas*), *R26*^CMER/+^ (Myc) and *TetO-HRas; Myh6-tTA; R26* ^CMER/+^ (Myc/*HRas*) mice 4 weeks post withdrawal of doxycycline and 24 h post administration of tamoxifen. Representative images based on analysis of at least 3 independent mice. Right- quantification of CC3 positive cardiomyocytes in hearts isolated from control (*R26*^+/+^*; TetO-HRas, R26*^+/+^*; Myh6-tTA* or *R26*^+/+^, black, *n* = 8), *TetO-HRas; Myh6-tTA; R26*^+/+^ (*HRas*, red, *n* = 4), *R26*^CMER/+^ (Myc, blue, *n* = 4) and *TetO-HRas; Myh6-tTA; R26* ^CMER/+^ (*Myc/HRas*, purple, *n* = 3) mice 4 weeks post withdrawal of doxycycline and 24 h post administration of tamoxifen. Bars are 100 μm Representative images based on analysis of 5 images per mouse; error bars show s.e.m. One-Way ANOVA with multiple comparisons test; Myc/HRas *vs* control: *p* = 0.0003, Myc/HRas vs Myc: *P* = 0.0006, Myc/HRas vs HRas*; Myh6-tTA; R26*^+/+^: *p* = 0.0295. **(B)** Left- immunofluorescent staining of heart from Control and *Myh6-Cre*; *R26*^LSL– CMER/+^ (Myc/Cyclin T1) mice 4 weeks post systemic infection with an AAV-Cyclin T1 adeno-associated virus and 48 post administration of tamoxifen (tam) at adulthood. Top: Control is *R26*^+/+^, cleaved caspase 3 (green) and cardiac troponin (pink), Bottom: Control is *Myh6-Cre*; *R26*^LSL– CMER/+^ without tam treatment. TUNEL (green). Representative images based on analysis of 5 independent mice. Right- quantification of TUNEL positive cells in hearts isolated from *R26*^+/+^ (WT) mice infected with AAV-LacZ and or AAV-Cyclin T1 and *Myh6-Cre*; *R26*^LSL– CMER/+^ (Myc) mice 4 weeks post systemic infection with an adeno-associated virus and 48 post administration of tamoxifen (tam). Bars are 100 μm. Representative images based on analysis of 5 images per mouse; error bars show s.e.m. Thymus is shown as a positive control. One-Way ANOVA with multiple comparisons test. ns = not significant. **(C)** Venn diagram of the overlap of the GSEA hallmark signatures obtained from the upregulated gene expression profile of Myc/HRas expressing hearts or Myc/Cyclin T1 expressing hearts. Adult mouse hearts were either isolated 4 weeks post systemic infection with an adeno associated virus encoding *Ccnt1* (*AAV9-Ccnt1*) compared to control and heart from *TetO-HRas; Myh6-tTA; R26* ^CMER/+^ (*n* = 3) 4 weeks after HRas^G12D^ expression and 4 h post Myc activation compared to control, as determined by RNA sequencing (FDR < 0.05 and abs(log2FC) > 0.5). The significant non-overlapping GSEA hallmark signatures are shown below.

It has been previously noted that HRas^G12V^ hypertrophic hearts display increased levels of cell death, mononuclear cell infiltration, and damage that ultimately lead to heart failure (30). We observed an increase in the presence of mononuclear cell infiltration such as CD206 positive macrophages (Figure 1A) and the apoptosis marker cleaved caspase 3 (Figure 5A). We also observed increased cleaved caspase 3 when elevated HRas^G12V^ expression was combined with MycER^T2^ activation (Figure 5A) and an apoptotic gene expression signature was present (Supplementary Figure 1D). Therefore, longer-term analysis and cardiomyocyte dissociation and counting of these mice hearts were not possible. Although elevated levels of Myc expression are associated with apoptosis in some tissues (43) we did not observe apoptosis in hearts expressing activated MycER^T2^ in the absence of HRas^G12V^ (Figure 5A). Using a different system, we next determined if the cell death observed with HRas^G12V^ and Myc together was recapitulated by co-activation of MycER^T2^ and Cyclin T1. We infected, *R26*^CMER/+^, and *R26*^LSL–CMER/+^;*Myh6Cre* mice with cardiac-specific (cTnT promotor-dependent) overexpression of Cyclin T1 using *AAV9-cTnT-Ccnt1* or a control β-galactosidase virus (*AAV9-cTnT-LacZ*). In contrast, to control mice, MycER^T2^ activation in the presence of Cyclin T1 increased cardiomyocyte proliferation as previously described (11), increased heart weight to tibia size ratio, increased number of Ki67 and p-H3 positive cardiomyocyte nuclei (Supplementary Figure 4) (11). However, we found no evidence of apoptosis (cleaved caspase 3) in cardiomyocytes in hearts overexpressing Myc and Cyclin T1 (Figure 5B). To confirm negativity we also performed Terminal deoxynucleotidyl transferase dUTP nick end labelling (TUNEL) for cell death which also indicated that elevated Myc and Cyclin T1 activity does not induce cardiomyocyte cell death within 48 h (Figure 5B). While the method for overexpressing Cyclin T1 differed, we compared the upregulated gene expression profile of Myc/HRas expressing hearts and Myc/Cyclin T1 expressing hearts and found the GSEA hallmark signatures vastly overlapped. Myc/Ras expressing hearts showed additional gene set enrichment for apoptosis (including the pro-apoptotic proteins Bak and Bok), epithelial mesenchymal transition and inflammatory response (Figure 5C and Supplementary Table 4). The only GSEA pathway that was enriched in Myc/Cyclin T1 expressing hearts was Wnt/Beta Catenin. These data indicate that cell death is specific to HRas^G12V^ activation, not simply an inevitable consequence of enforced endogenous cardiomyocyte proliferation.

## Discussion

Myc is a transcription factor that serves as a pivotal instructor of tissue regeneration following injury in a regenerative organ (13), however, in the heart both Myc transcription and Myc-driven transcription are attenuated. When Myc is acutely overexpressed in the heart it competently binds to DNA but only drives a limited transcriptional programme, and cell-cycle progression is not observed within 48 h (11). Protracted Myc expression in cardiomyocytes eventually leads to DNA synthesis, and myocyte hypertrophy but not to cardiomyocyte cytokinesis (15). We have previously shown that the ability of Myc to drive all the transcriptional programmes necessary for cell division in cardiomyocytes depends on the level of P-TEFb (11). We and others have shown that the level of P-TEFb is dependent on the level of Cyclin T1 and elevated Cyclin T1 leads to a corresponding increase in CDK9 and phosphorylated RNA PolII (11, 25, 26). Since HRas^G12V^ upregulates P-TEFb in cardiomyocytes (31) resulting in cardiac muscle hypertrophy, we hypothesised that Myc and Ras would co-operate to drive cardiomyocyte proliferation.

We employed a previously described mouse model of HRas^G12V^ overexpression in which oncogenic *HRas*^G12V^ is under the control of a tetracycline response element (*TetO-HRas)* harbouring a cardiomyocyte-specific reverse tetracycline transactivator (*Myh6-tTA)*. Withdrawal of doxycycline at 4 weeks of age leads to cardiomyocyte-restricted HRas^G12V^ overexpression and pathogenic myocardial hypertrophy. Cessation of HRas^G12V^ activity after induction of hypertrophy leads to hypertrophy resolution (30). We confirmed that long-term overexpression of HRas^G12V^ in cardiomyocytes led to increased HRas signalling and cardiomyocyte hypertrophy as previously reported (29, 30, 44–46). Four weeks after HRas^G12V^ expression higher levels of CDK9, Cyclin T1, and phosphorylated RNA Pol II in HRas^G12V^ were observed compared to control animals, confirming that long-term HRas^G12V^ expression upregulates the transcriptional elongation machinery. The exact mechanism of increasing P-TEFb activity by Ras is not known, however, recently the downstream Ras effector AP-1 (heterodimeric transcription factors comprising members of the Fos and Jun families) has been shown to be critical for both Zebrafish and *Xenopus tropicalis* heart regeneration (47, 48). Fosl1 plays an essential role in cardiomyocyte proliferation by interacting with JunB and binding the Ccnt1 promoter, increasing the expression of Cyclin T1 (48). Here we show that HRas^G12V^ overexpression in cardiomyocytes leads to increased Fosl1 and an increase in Cyclin T1 protein levels. We have previously demonstrated that Myc co-operates with elevated Cyclin T1 expression to drive cardiomyocyte proliferation *in vivo* (10), therefore, we hypothesised that Myc and HRas would also co-operate in cardiomyocyte proliferation. In the presence, but not the absence, of HRas^G12V^, Myc activation competently elicited transcriptional programmes involved in cell growth, biogenesis, and metabolism that led to a cardiomyocyte proliferative response. Overlap of the genes altered in Myc and HRas activated hearts with those altered during regeneration of P1 neonatal cardiomyocytes indicated a strong correlation, suggesting that common regenerative transcriptional pathways are activated.

Quantification of cardiomyocyte division is notoriously challenging as cell-cycle re-entry does not necessarily lead to cell division (49–51), and the cell death observed in the HRas model prohibited long-term experimentation and cardiomyocyte number estimation by dissociation techniques. Ki67 is expressed during all active phases of the cell cycle while p-H3 expression begins in G2 and is present throughout mitosis. These markers relay no information regarding cytokinesis and hence they are technically markers of cell cycling and not necessarily proliferation. Here, we observe both Ki67 and p-H3 expression in cardiomyocytes (Figure 4D) demonstrating that combined Myc and HRas overexpression drive cardiomyocytes into the cell cycle. Aurora B kinase is expressed during G2, anaphase, metaphase, telophase, and cytokinesis, where it is detected within the mid-body and can be used as a marker of cardiomyocyte division. However, it is present in an asymmetrical location within cardiomyocytes undergoing bi-nucleation (49) and caution is needed when interpreting staining patterns (50). Here we observed Aurora B kinase localisation in cardiomyocytes in prophase, metaphase, anaphase/telophase and symmetrical mid-bodies. Mitotic kinesin-like protein 1 (Mklp1) is an additional marker of cardiomyocyte cytokinesis where it accumulates at the cleavage furrow (52, 53). Here we observed Mklp1 staining at the cleavage furrow of cardiomyocytes in Myc/HRas overexpressing hearts. While these data provide some evidence that Myc and HRas together drive cardiomyocytes’ entry into the cell cycle with positivity for markers of cytokinesis, productive cell division cannot be formally confirmed without direct cardiomyocyte counting.

In healthy adult mammalian heart apoptosis is rare with only 0.01–0.001% TUNEL-positive cardiomyocytes observed. This rises to 2–12% apoptotic cardiomyocytes in MI ischaemia and reperfusion injury (54–56). Apoptotic death is also a common feature of cardiomyocytes driven into the cycle by overexpression of some cell cycle regulators such as E2F1, CDK1, and CCNB (8, 57). Paradoxically oncogenes, particularly Myc and Ras, promote both pro-proliferative and pro-apoptotic signals, and the cell fate outcome depends very much on the cell type and context (58). In cardiomyocytes, PI3K and ERK activation downstream of HRas has strong anti-apoptotic effects, promoting cardiomyocyte survival in pressure-overload hypertrophy and heart failure (59). However, hypertrophic cardiomyopathy resulting from HRas mutation associated with chronic constitutively activated Ras/Raf/MEK/ERK pathway and pathological hypertrophy leads to apoptosis (60), similar to the phenotype seen in *TetO-HRas; Myh6-tTA* mice used in this study. We show that MycER expression alone did not lead to cell death in agreement with similar models (15). However, prolonged/persistent ectopic HRas^G12V^ signalling leads to apoptotic cell death, which was exacerbated by transient Myc signalling. Transcriptional profiling indicated Myc/HRas hearts expressed increased levels of the pro-apototic factors Bak, Bok and enhanced inflammatory response, which may be promoting apoptosis. These data indicate endogenous cardiac regeneration is unlikely to be effective with the combination of Myc and HRas. More importantly, the data suggest patients with elevation in Ras signalling in hypertrophic hearts may not benefit or may be harmed, from driving endogenous cardiomyocyte regeneration via activation of cell cycle genes such as Myc. Here, we activated HRas for 4 weeks, further studies are required to establish whether a shorter period of HRas expression results in similar transcriptional changes and proliferation without hypertrophy and apoptosis.

More encouragingly, elevated Cyclin T1 expression, absent of HRas activation, co-operated with activated Myc to drive cardiomyocyte proliferation in the absence of apoptosis, suggesting that co-expression of Cyclin T1 and Myc may be a therapeutically tractable approach for heart regeneration after injury. Myc/Cyclin T1 hearts expressed an increased Wnt/Beta catenin signature, which has recently been shown to be cardioprotective in an adult mammalian setting (61), deciphering the key protective and destructive pathways will be key to translating these findings. As with all genes able to drive proliferation in cardiomyocytes the expression must be tightly regulated and localised. Promising modes of delivery are being developed including cell-specific viral expression vectors and transient modified mRNA technologies.

## Study limitations

HRas^G12V^ is restricted to the cardiomyocytes in the *TetO-HRas; Myh6-tTA; R26*^CMER/+^ mice, but Myc is expressed across the whole animal, so care must be taken to draw conclusions from the bulk transcriptional data because the interaction between Myc activated non-myocytes and cardiomyocytes are possible. The different systems used to overexpress HRas (*TetO-HRas; Myh6-tTA; R26*^CMER/+^ mice) and Cyclin T1 (*AAV9-cTnT-Ccnt1*) mean the apoptosis and proliferation efficiencies between these groups cannot be directly compared.

## Data availability statement

All datasets generated and used in this study, have been deposited in ArrayExpress (www.ebi.ac.uk/arrayexpress) under accession codes: E-MTAB-7595, E-MTAB-8462, and E-MTAB-7636. Further information and requests for resources and reagents should be directed to, and will be fulfilled, by CW, chw39@cam.ac.uk.

## Ethics statement

All experimental procedures received ethical approval and were conducted in accordance with the Home Office UK guidelines, under project licences 70/7586 and 80/2396 (GE) and PP2054013 (CW) that were evaluated and approved by the Animal Welfare and Ethical Review Body at the University of Cambridge.

## Author contributions

MB and CW conceptualised the study. AB, MB, GQ-R, CA, and LT performed the experimental work. GQ-R and JS performed the sequencing analysis. AB, MB, and CW wrote the manuscript. GE and TL edited the manuscript. GE, JH, and CW obtained the funding. All authors contributed to the article and approved the submitted version.

## Footnotes

1
http://www.gsea-msigdb.org/gsea/index.jsp

2
www.lucidchart.com

3
https://software.broadinstitute.org/morpheus
