# Supplementary material for: HRas and Myc synergistically induce cell cycle progression and apoptosis of murine cardiomyocytes
Source: Front Cardiovasc Med. 2022 Oct 20;9:948281. doi: 10.3389/fcvm.2022.948281 (PMC9630352; doi:10.3389/fcvm.2022.948281)
Supplement: Supplementary file 1 [file Data_Sheet_1.pdf]

HRas and Myc synergistically induce cell cycle progression and apoptosis of murine cardiomyocytes

Aleksandra Boikova, Megan J Bywater, Gregory A Quaife-Ryan, Jasmin Straube, Lucy Thompson, Camilla Ascanelli, Trevor D Littlewood, Gerard I Evan, James E Hudson, Catherine H Wilson.

Supplemental Data and Figures

Supplemental Figure 1

(A) Quantification of individual cardiomyocyte cell area from immunofluorescent staining of cardiac troponin and WGA in the heart isolated from control (*$R26^{+/+}$; TetO-HRas, $R26^{+/+}$; Myh6-tTA* or *$R26^{+/+}$*, black, n=423), *TetO-HRas; Myh6-tTA; $R26^{+/+}$* (*HRas*, red, n=248), *$R26^{CMER/+}$* (Myc, blue, n=520) and *TetO-HRas; Myh6-tTA; $R26^{CMER/+}$* (*Myc/HRas,* purple, n=248) mice 4 weeks post withdrawal of doxycycline and 24 hours post administration of tamoxifen. Cells area was estimated in ImageJ software. One Way ANOVA with multiple comparisons test: P<0.0001****, P=0.016*. (B) Quantification of protein expression from western blots shown in Figure 1C showing relative expression over Gapdh. (C) Quantification of protein expression from western blots shown in Figure 2B showing relative expression over Gapdh. Unpaired T-test: Control vs phosphorylated RNA Polymerase II (p-Rpb1(S2)) P=0.0046, Cyclin T1 P=0.046 and CDK9 P=0.0048

(D) Venn diagram of overlap in the number of genes showing an increase in expression in response to supraphysiological Myc expression. The most significant GSEA Hallmarks gene lists that overlap are shown. Comparisons included the liver of *$R26^{CMER/+}$* (Myc liver, *n*=3) compared to wild type. The adult mouse heart isolated 4 weeks post systemic infection with an adeno associated virus encoding *Ccnt1* (*AAV9-Ccnt1*) compared to control and heart from *TetO-HRas; Myh6-tTA; $R26^{CMER/+}$* (n=3) four weeks after HrasG12V expression and 4 hours post $MycER^{T2}$ activation. compared to control, as determined by RNA sequencing (FDR<0.05 and abs(log2FC)>0.5).

Supplemental Figure 2

(A) Metabolic genes (FDR < 0.05) isolated from neonatal (MIP1.Myo) and adult (MIP56.Myo) cardiomyocytes isolated from myocardial infarction-operated hearts (n=4), adult *TetO-HRas; Myh6-tTA; $R26^{+/+}$* (*HRas*) and adult *TetO-HRas; Myh6-tTA;*

R26 $^{CMER/+}$ (*Myc/HRas*) hearts and overlayed them with the KEGG metabolic pathway. Active pathways are labelled in black.

(B) Gene set enrichment of E2F targets and G2M checkpoint gene lists in comparison to differential gene expression observed between *TetO-HRas; Myh6-tTA; R26* $^{CMER/+}$ mice (*Myc/HRas, n*=3) and *TetO-HRas; Myh6-tTA; R26* $^{+/+}$ mice (*n*=3) at 4 hours post administration of 4-OHT.

Supplemental Figure 3

Left- Quantification and of cell cycle phase of Aurora B kinase positive cardiomyocytes in *TetO-HRas; Myh6-tTA; R26* $^{CMER/+}$ (n=3) four weeks after HrasG12V expression and 24 hours post MycER^{T2} activation. Data points represent percent ABK positive in each phase from each image assessed. Right- Representative immunofluorescent staining of quantified cell cycle phases, cardiac troponin (red) and Aurora B Kinase (green) in the heart of *TetO-HRas; Myh6-tTA; R26* $^{CMER/+}$ (Myc/*HRas*) mice 24 hours post administration of tamoxifen. Images based on analysis of at least 3 independent mice.

Supplemental Figure 4

(A) The weight (mg) of hearts isolated from *R26* $^{+/+}$ (WT) mice infected with AAV-LacZ and or AAV-Cyclin T1 and *Myh6-Cre*; *R26* $^{LSL-CMER/+}$ (Myc) mice 4 weeks post systemic infection with an adeno-associated virus and 48 post administration of tamoxifen (tam) at adulthood, expressed as fold change over the length (mm) of a tibia isolated from the same mouse. Mean and s.e.m shown. One-way ANOVA with Tukey's multiple comparisons test. Replicate samples are derived from independent mice. Values for p shown.

(B) Quantification of p-H3-positive cardiomyocyte nuclei percentage in *R26* $^{+/+}$ (WT) mice infected with AAV-LacZ and *Myh6-Cre*; *R26* $^{LSL-CMER/+}$ (Myc) mice 4 weeks post systemic infection with an adeno-associated virus and 48 post administration of tamoxifen (tam). Means are taken from five images per mouse; Mean and s.e.m shown. Kruskal-Wallis with Dunn's multiple comparisons. Values for P shown.

(C) Quantification of Ki67-positive cardiomyocyte nuclei percentage in *R26*$^{+/+}$ (WT) mice infected with AAV-LacZ and *Myh6-Cre*; *R26*$^{LSL\text{-}CMER/+}$ (Myc) mice 4 weeks post systemic infection with an adeno-associated virus and 48 post administration of tamoxifen (tam). Means are taken from five images per mouse; Mean and s.e.m shown. Kruskal-Wallis with Dunn’s multiple comparisons. Values for P shown.

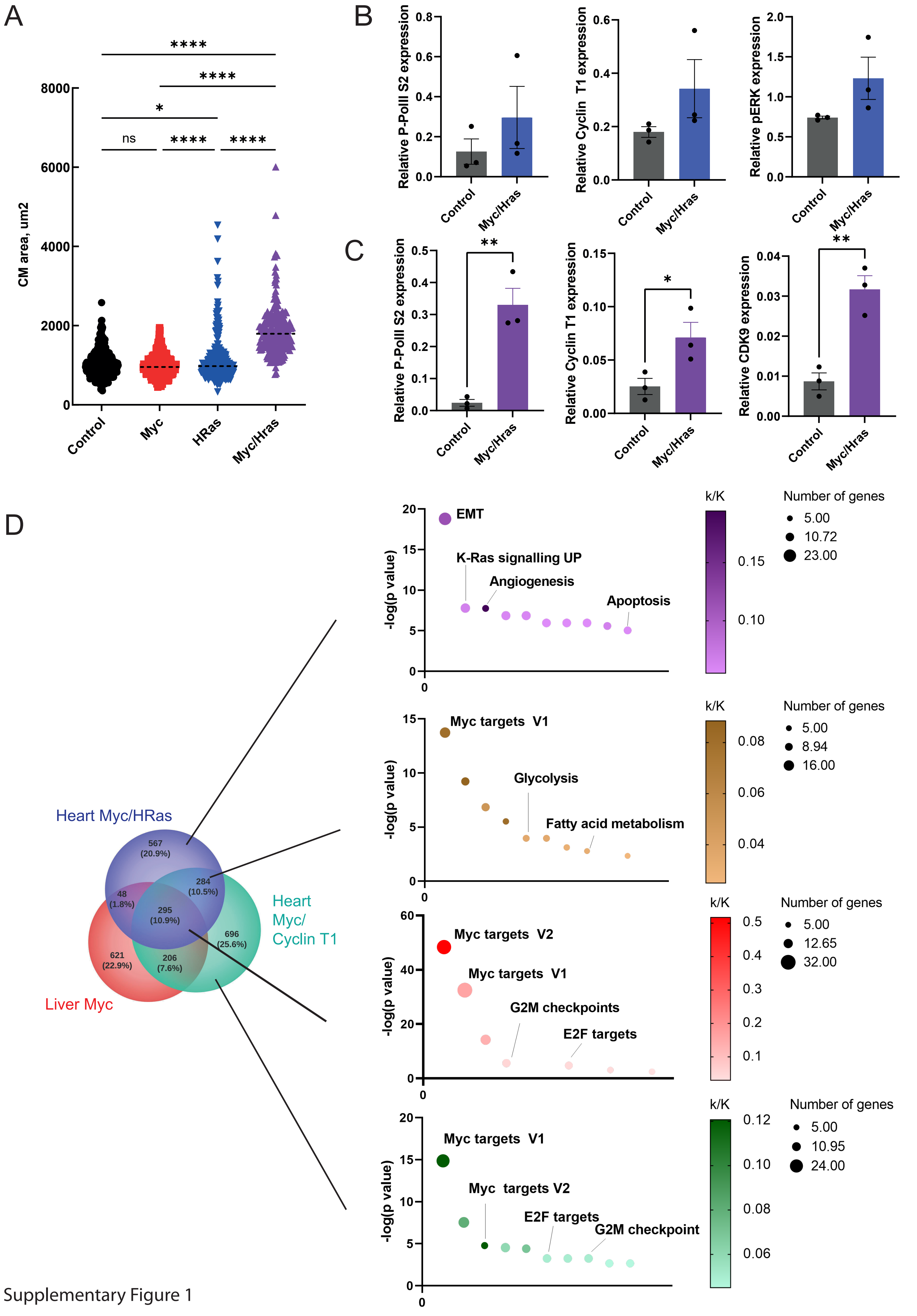

Supplementary Figure 1

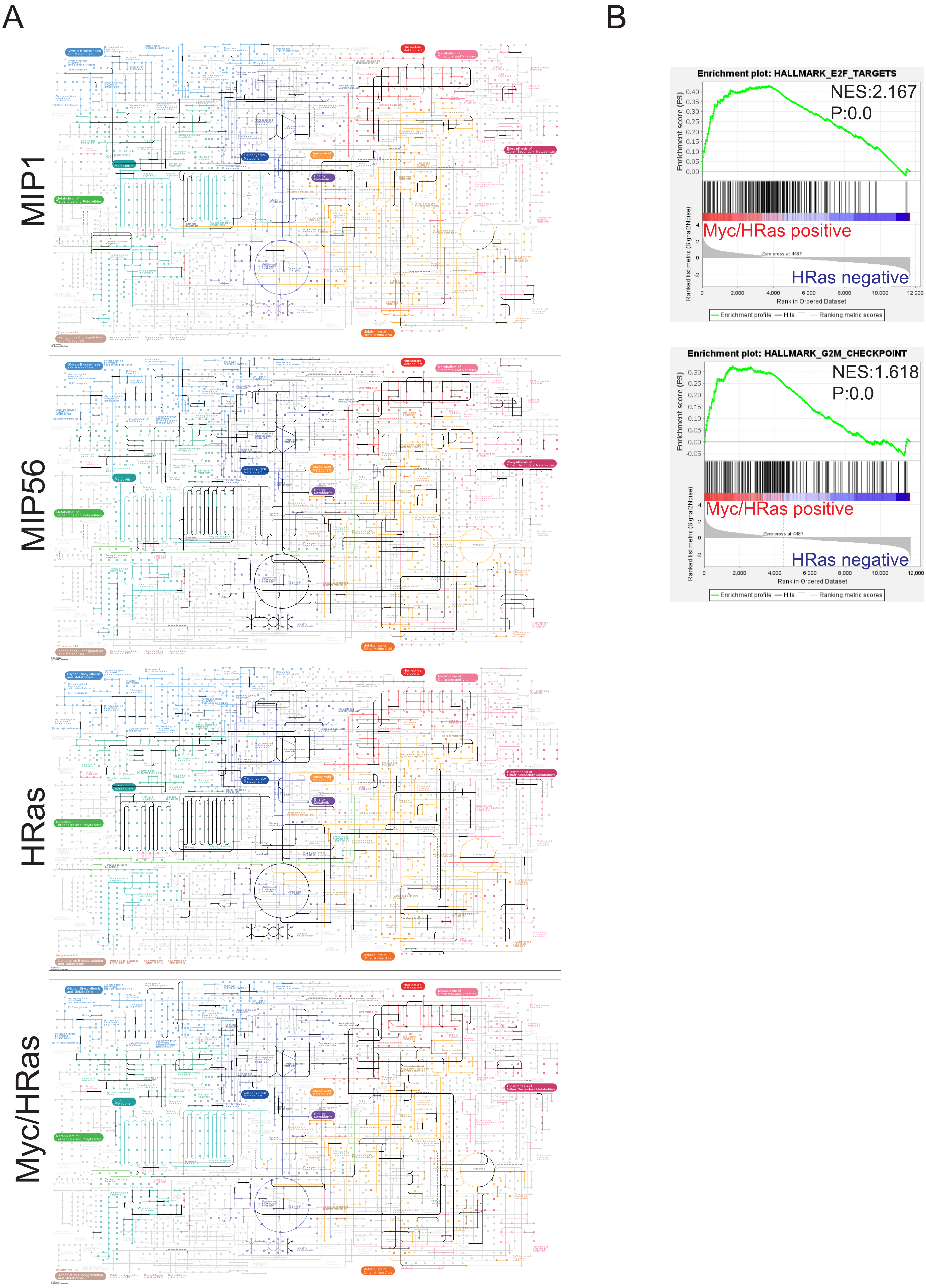

Supplementary Figure 2

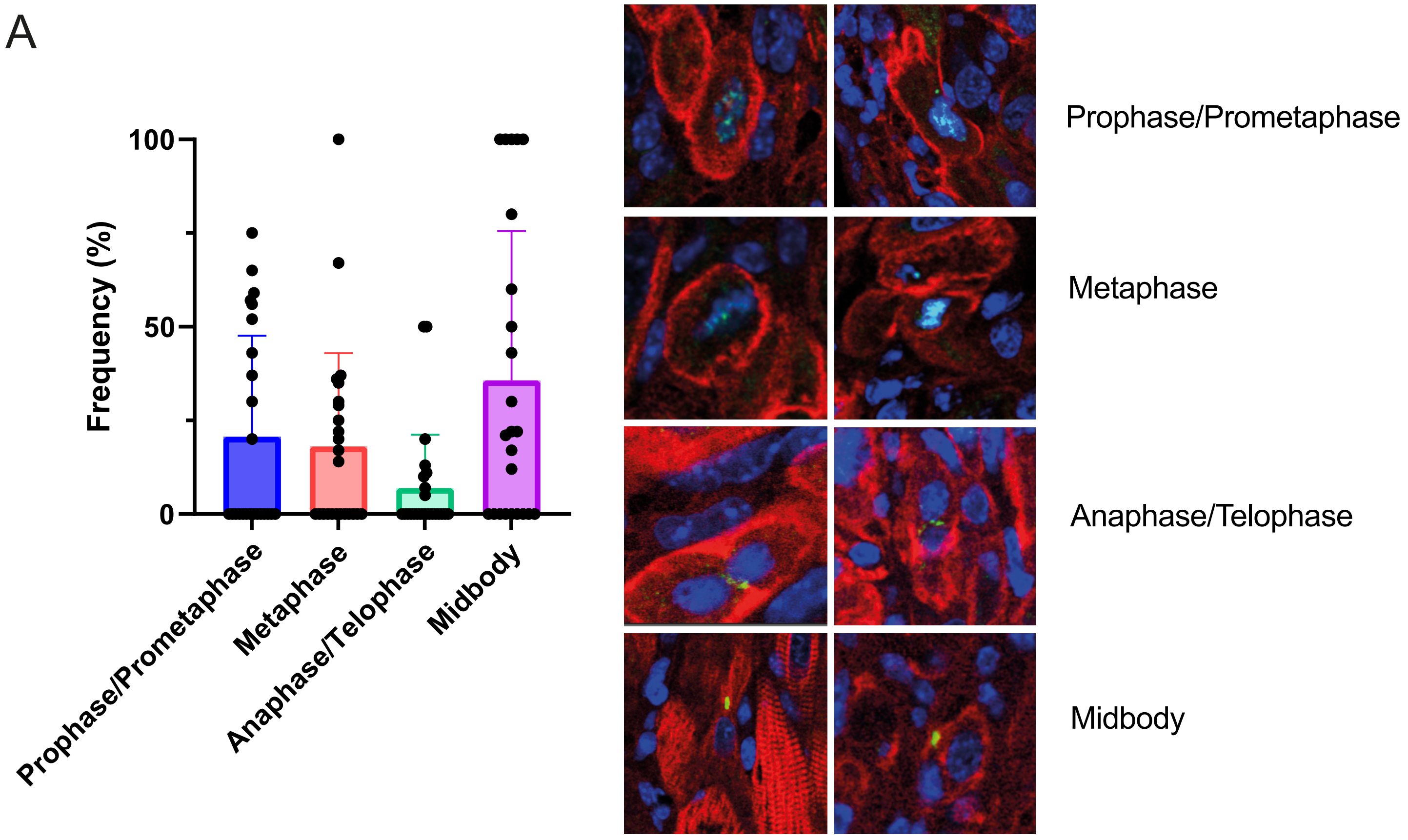

Supplementary Figure 3

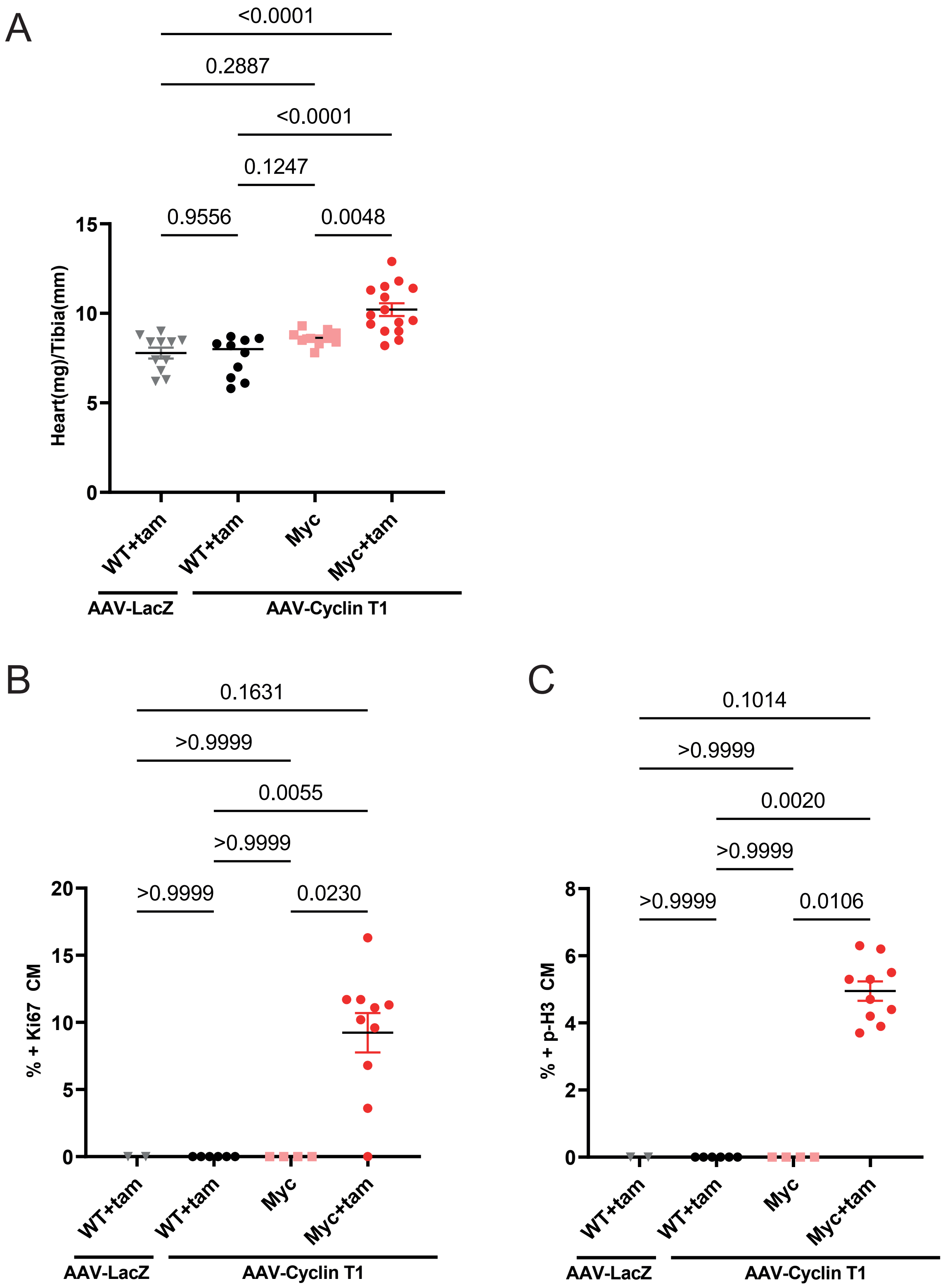

Supplementary Figure 4
